# Numerical Analysis in Double-Sided Polishing: Mechanism Exploration of Edge Roll-Off

**DOI:** 10.3390/ma17194761

**Published:** 2024-09-27

**Authors:** Jiayu Chen, Yiran Liu, Ding Wang, Wenjie Yu, Lei Zhu

**Affiliations:** 1 School of Materials and Chemistry, University of Shanghai for Science and Technology, Shanghai 200093, China; gary@mail.sim.ac.cn (J.C.); wangding@usst.edu.cn (D.W.); 2 State Key Laboratory of Materials for Integrated Circuits, Shanghai Institute of Microsystem and Information Technology, Chinese Academy of Sciences, Shanghai 200050, China; casan@mail.sim.ac.cn; 3 Shanghai Institute of IC Materials, Shanghai 200050, China; yiran.liu@sicm.com.cn

**Keywords:** semiconductor substrate material, double-sided polishing, edge roll-off, finite element analysis, contact stress

## Abstract

Understanding the mechanism of stress concentration effects on the surface of semiconductor substrate materials—silicon wafers—in Double-Sided Polishing (DSP) is particularly important for improving polishing quality. In this study, a two-dimensional finite element model is established to study the effect of contact state and stress concentration during polishing on edge roll-off (ERO) and polishing rate uniformity. The variation in this contact state is influenced by changes in wafer thickness and the gap between it and the carrier. The model is validated by experiments and helps to further analyze and interpret the experimental results, identifying six stages of contact states during the polishing process. The research indicates that the phenomenon of stress concentration at the edge of a wafer is caused by the pads creating a large amount of compression at the edge of the wafer. Additionally, there appears to be a threshold value during the polishing process, below which the stress concentration on the wafer changes, thereby altering the magnitude of edge roll-off and, ultimately, affecting overall flatness. This study provides a basis for optimizing the process design.

## 1. Introduction

**Figure 1:**
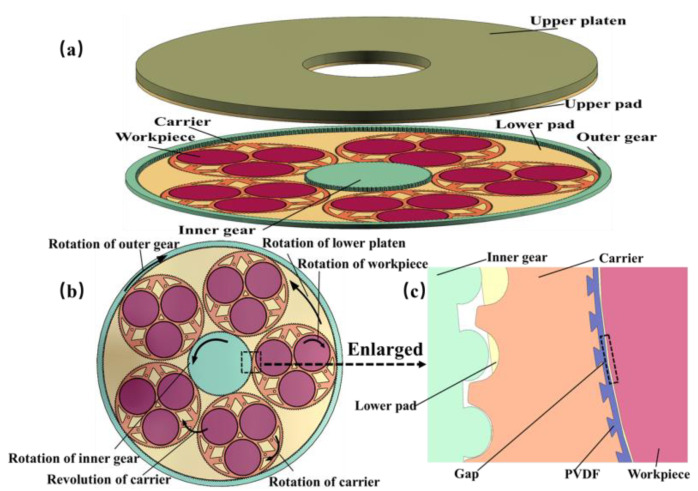
Schematic diagram of DSP. (**a**) Overall structure; (**b**) top view; and (**c**) wafer edge structure.

DSP has become an indispensable step in chip substrate processing, owing to its efficiency in material removal from both the upper and lower surfaces compared with single-sided polishing (SSP), which brings a more complex stress situation [1,2,3,4,5]. In DSP, the silicon wafer is placed in the carrier, which is “sandwiched” by upper and lower pads. Controlled by the inner and outer gears, the wafer rotates and revolves within the carrier, while the upper pad and the lower pad rotate respectively, as shown in Figure 1 [6,7]. The wafer contacts with pad and slurry particles under pressure, thus generating friction to remove material [8].

**Figure 2:**
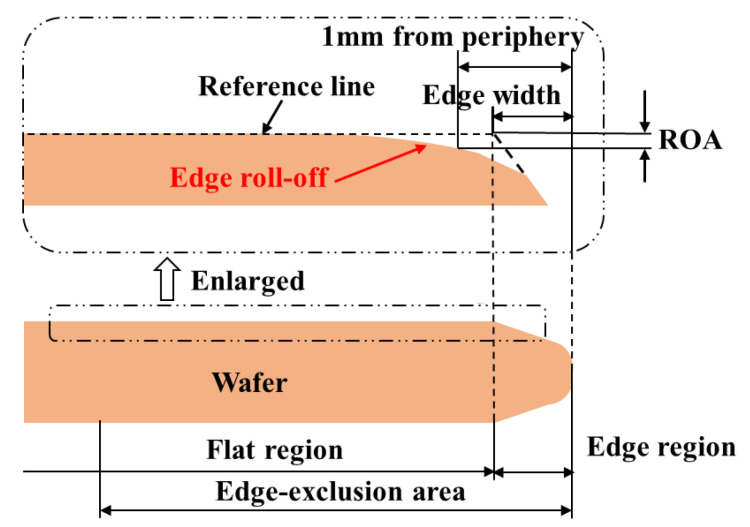
Schematic view of wafer surface profile near the edge and wafer edge roll-off. (Reprinted from [14], Akira Fukuda, Tetsuo Fukuda, Akira Fukunaga, Manabu Tsujimura, Influence of Wafer Edge Geometry on Removal Rate Profile in Chemical Mechanical Polishing: Wafer Edge Roll-Off and Notch, Page No.1, Copyright (2012), with permission from Copyright Clearance Center).

During the polishing process, the manifestation of a significant reduction in wafer edge thickness due to irregularities in wafer edge polishing is called “ERO”, which is very common in DSP [9,10,11]. This phenomenon detrimentally impacts the wafer edge planarity, thus prompting the imperative inquiry into the factors leading to edge roll-off, as shown in Figure 2 [12,13].

In the polishing process, the material removal rate (MRR) is proportional to the normal pressure according to Preston’s equation [15]:

(1) $$MRR=C_{p}\times P\times V$$

where P is the normal pressure, V is the relative velocity, and $C_{p}$ is the Preston’s constant. It has been reported that edge roll-off mainly results from stress concentration at the silicon wafer edge [14,16]. As the pad exhibits irregular deformation, there is an uneven distribution of stress on the wafer, especially at the edge, leading to edge roll-off [17,18,19]. However, the polishing process and pad displacement could not be observed as the equipment is non-transparent. In recent years, simulations have helped to solve the limitations in experiments and explain some influencing factors from the perspective of mechanism. Therefore, it is necessary to explain edge roll-off in polishing from a solid mechanics point of view through simulations.

Some studies have shown that edge roll-off in chemical mechanical polishing can have a significant impact on overall flatness [6,20,21,22]. Hence, it is crucial for wafer uniformity to understand the formation mechanism of edge roll-off and, thereby, improve ERO [22,23]. Satake U et al. developed a three-layer pad to obtain overall flatness while reducing the amount of ERO [24]. Ren L et al. improved edge roll-off during large planar optics polishing by using extension blocks to extend the edge [25]. Pan B et al. proposed double-sided polishing with fixed abrasives to reduce the depth and width of edge roll-off on thin copper substrates [26]. Miyake T et al. investigated the influence of polishing pressure on edge shape and emphasized the importance of polishing pressure for achieving high flatness of the workpiece edge [27]. Miyake T and Satake U et al. also studied the mechanism of edge roll-off based on the viscoelasticity of pads, offering theoretical guidance for reducing edge roll-off on wafers [23,28]. Lo S P et al. investigated the effects of the gap between the retaining ring and the wafer in single-sided polishing (SSP) and the ratio of the load applied on the retaining ring and wafer. Wafer nonuniformity was improved when the gap and load were designed within a certain range to improve von Mises stress distribution [29]. Yu TK and Yu CC have shown in previous studies that the stress on the wafer surface comes from the positive pressure exerted by the upper pad on the one hand and the shear stress exerted by the pad on the wafer itself during the rotation process on the other hand [30]. Past research on polishing mainly improves edge roll-off by directly adopting different consumable materials to reduce stress concentration, while it pays little attention to analyzing the basic solid mechanism of edge roll-off at different stages during the polishing process. The motion process of the wafer in DSP has more non-determinism, which is manifested by the gap between the wafer and the carrier being in a constant state of change. This makes the contact state also more variable. Therefore, to improve the edge roll-off phenomenon of large-sized wafers, the polishing process of DSP needs to be analyzed theoretically.

In this paper, a finite element model is established to study the effect of thickness differences between a wafer and a carrier at different DSP stages. The results show that as the wafer thickness decreases, the contact state undergoes six stages, leading to varying degrees of stress distribution nonuniformity and edge roll-off. This study provides insights on the edge roll-off mechanism in DSP and offers research direction for carrier and wafer polishing thickness difference control standards.

## 2. Materials and Methods

### 2.1. Model Geometry

**Figure 3:**
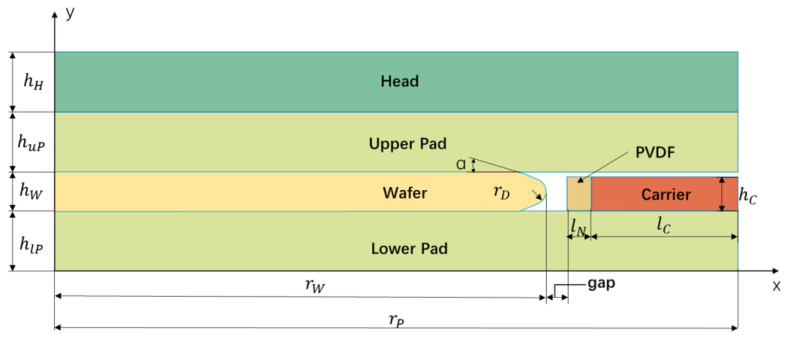
Definition of the dimensions of the model.

In this study, the software employed is the Mechanical module in ANSYS 2024 R1, which is specifically designed for conducting structural analysis and computational simulations. The hardware utilized to support the simulations consists of a server equipped with a Gold 6248R CPU, which features 48 cores and 48 threads, backed by 767 GB of RAM. In order to focus on the edge roll-off study edge, the model is simplified to being two-dimensional, and the analysis is confined within 0–25 mm at the silicon wafer edge, as shown in Figure 3. The meaning of each parameter symbol is shown in Table 1.

### 2.2. Governing Equation

In this model, the structural mechanics of DSP are described as follows:

(1) Equilibrium equations [31]:
  (2) $$\left\{\begin{array}{c} \frac{\partial \sigma_{x}}{\partial x}+\frac{\partial \tau_{xy}}{\partial y}+f_{x}=\rho \frac{\partial^{2}u}{\partial t^{2}}\\ \frac{\partial \tau_{xy}}{\partial x}+\frac{\partial \sigma_{y}}{\partial y}+f_{y}=\rho \frac{\partial^{2}v}{\partial t^{2}} \end{array}\right.$$
  where $f_{x}$ is the volumetric force component in the x-direction, and ρ is the density.
(2) Constitutive equation [32]:
  (3) $$\left[K\right]\left\{\delta \right\}=\{Q\}$$
  (4) $$\left[K\right]=\sum_{all\;elements}{∭}_{V}{\left[B\right]}^{T}\left[D^{e}\right]\left[B\right]dV$$
  (5) $$\left[Q\right]=\sum_{surface}\underset{V}{∭}{\left[N\right]}^{T}\left\{F_{b}\right\}dV+\iint_{S}{\left[N\right]}^{T}\{T_{d}\}dS$$
  where $\left[K\right]$ is the elastic stiffness matrix, $\left\{\delta \right\}$ is the nodal displacement vector, {Q} is the nodal force vector, $\left[B\right]$ is the strain-displacement matrix, $\left[D^{e}\right]$ is the elastic stress–strain relation matrix, [N] is the shape function matrix, $\left\{F_{b}\right\}$ is the body force, $\left\{T_{d}\right\}$ is the surface traction, V is the volume, and S is the prescribed surface exerted by traction.

### 2.3. Boundary Conditions

This paper focuses on the effects of material and structural changes on the wafer’s stress during the polishing process, providing a mechanistic explanation for the edge roll-off phenomenon. To minimize the coupling effects of excessive variables, assumptions and settings are made to simplify the boundary conditions. The boundary conditions for the finite element model developed in this paper can be described as follows:

1. This paper employs a steady-state model to analyze stress distribution at each moment. The actual polishing process is transient, with contact conditions varying continuously due to the material properties and coupling environment during structural movement. The use of steady-state solutions in this paper significantly improves computational efficiency and reduces costs. Simulations represent ideal conditions, and the actual degradation effects in the process may be less severe than the results calculated in this study.
2. The ground surface of the lower pad is defined as a fixed support. A uniform load is applied to the polishing head. This method is consistent with the pressure application approach used in subsequent experiments. However, in the actual processes, some machines utilize zoned pressure adjustment. Therefore, simulations need to be configured to align with the specific operating conditions.
3. Bonded contact is adopted between the polishing head and the upper pad, as well as between the carrier and PVDF liner. Contact between the rest of the parts is set as frictional contact. Friction coefficients are shown in Table 2. In the actual process, the head is adhered to the pad using glue, and the lining material is engaged with the carrier. Although this may alter the material properties in the bonded area to some extent, the impact is minimal due to the relatively large dimensions in the normal direction. Additionally, while the friction coefficient is influenced by the pad material, the composition of the slurry, and the wafer’s morphology, its effect on the steady-state model is minor. The friction coefficient specified is applicable only to this model.

### 2.4. Grid Independence Verification

In the finite element simulation, the number of grids has a large impact on the simulation accuracy; thus, the grid independence verification is necessary. This paper focuses on the stress on the silicon wafer itself, so the grid independence is verified based on the maximum von Mises stress on the upper surface of the wafer.

**Figure 4:**
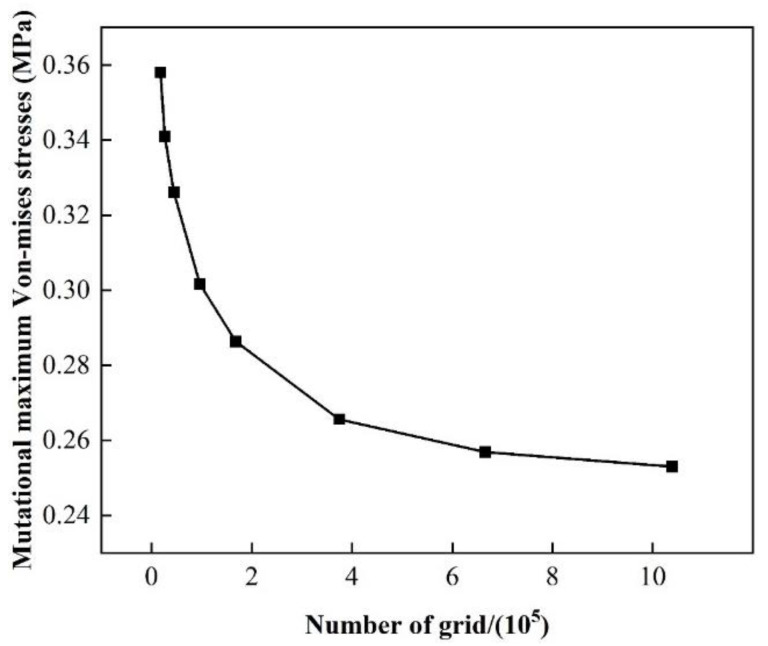
Effect of the number of grids on the von Mises stress.

As shown in Figure 4, the number of grids increases with 40,000 as the starting point. When the number of grids is lower than 160,000, the stress value is greatly influenced by the grids. As the number of meshes reaches 360,000, the stress value tends to stabilize. Therefore, the 370,000 grid model in this paper can both achieve high accuracy and efficiency. The variation in contact states can cause fluctuations in computation times, but the overall computation time ranges from 5 min and 40 s to 13 min and 10 s, which is within the expected outcomes.

### 2.5. Calculation Parameter

In the model studied in this paper, the material properties of each part in this model are listed in Table 3.

The simulation scheme and serial number in this paper are shown in Table 4 and Table 5. The thickness difference between the silicon wafer and the carrier during the polishing process is defined as Jut-out, as shown in Equation (6):

(6) $$\mathrm{Jut}-\mathrm{out}=h_{W}-h_{C}$$

## 3. Results and Discussion

### 3.1. Effect of Jut-Out on Edge Roll-Off

**Figure 5:**
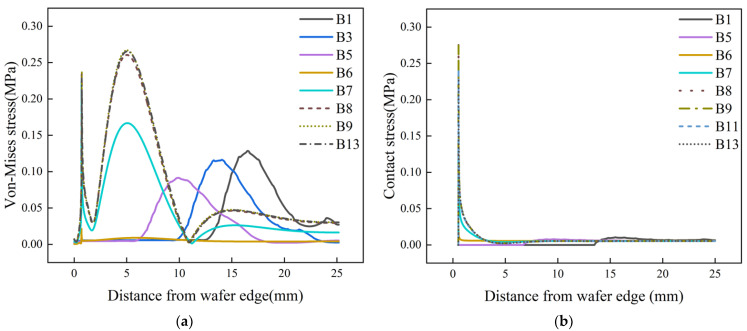
Stress on the upper surface of the wafer with different Jut-out (**a**) von Mises stress and (**b**) contact stress.

In order to study the contact state and stress concentration in a silicon wafer edge during the whole polishing process, simulations with different Jut-outs are carried out. In this paper, both contact stress and von Mises stress are used to evaluate edge roll-off [33]. Figure 5 shows stress distribution under different Jut-outs with a 0.5 mm gap.

In Figure 5a, it can be observed that when Jut-out > 2 μm, the stress curves with different Jut-out overlaps, and the von Mises stress peak is maximized. There are two peaks in the stress curve. The first stress peak is at 0.51 mm from the edge, which is in accordance with the edge of the wafer chamfer. Subsequently, the second stress peak with a wider range of fluctuation is generated at 5.06 mm from the edge. As the Jut-out decreases from 2 μm to 0 μm, the von Mises stress curve will tend to flatten, and the two maximum peaks begin to decrease. The stress peaks at the wafer chamfering still exist, but the peaks decrease compared to the last stage. The stress peak region at 5.06 mm significantly decreases, and the overall von Mises stress curve tends to be uniform. When Jut-out = 0 μm, the force on the upper surface of the wafer is most uniform during this polishing process. When Jut-out = −1 μm, the von Mises stress curve exhibits a stress peak at 10 mm from the edge. As the Jut-out continues to decrease, the stress peak position gradually shifts toward the wafer center, with a gradual increase in the stress peak value.

The conclusion can also be drawn from contact stress distribution, as shown in Figure 5b. When Jut-out > 2 μm, the maximum stress value occurs at the edge of the wafer. As the distance from the edge increases, the contact stress value gradually decreases and eventually tends to level off. When 0 μm ≤ Jut-out ≤ 2 μm, the edge of the wafer is still in the peak contact stress stage, but the peak stress value is reduced, and the contact stress is more uniform over the entire wafer surface. When Jut-out < 0 μm, the contact stress peak at the wafer edge disappears.

**Figure 6:**
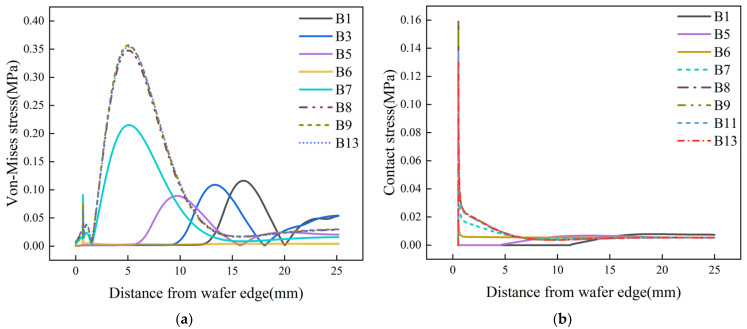
Stress on the lower surface of the wafer with different Jut-out (**a**) von Mises stress (**b**) and contact stress.

The von Mises stress on the lower surface, as shown in Figure 6a, differs only in the magnitude of the values, and the fluctuation trend is almost the same compared to the von Mises stress on the upper surface, as shown in Figure 5a. The contact stress on the lower surface, as shown in Figure 6b, has the same conclusion when compared with the contact stress on the upper surface, as shown in Figure 5b. It can be concluded that the edge roll-off locations on the upper and lower surfaces of the wafer are almost the same, but there is a difference in the magnitude of the edge roll-off.

**Figure 7:**
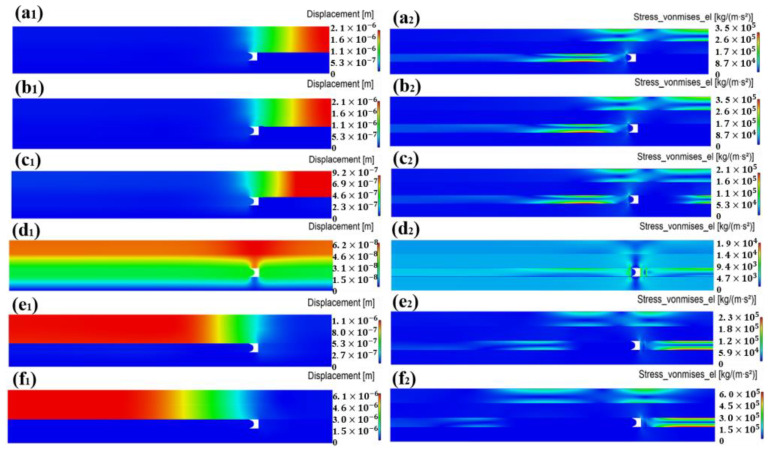
Displacement and stress diagrams for different contact states ((**a1**,**a2**): B13; (**b1**,**b2**): B9; (**c1**,**c2**): B7; (**d1**,**d2**): B6; (**e1**,**e2**): B5; and (**f1**,**f2**): B1).

**Figure 8:**
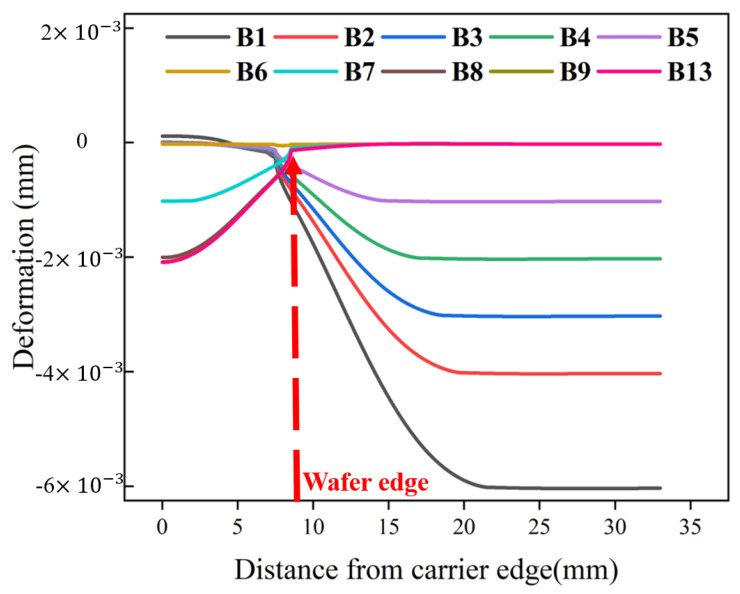
Deformation of the lower surface of the upper pad.

**Figure 9:**
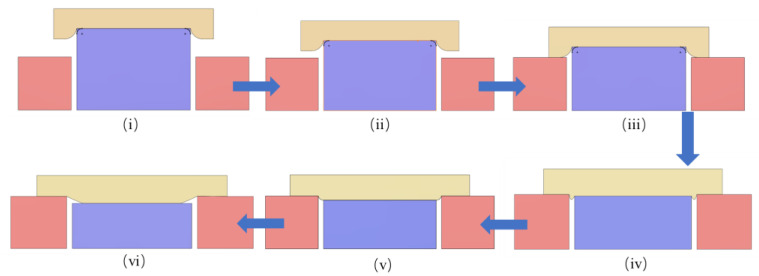
Schematic diagram of the change in contact state during the polishing process.

The above phenomenon can be explained by pad deformation and corresponding contact state analysis in Figure 7 and Figure 8. First, when Jut-out > 2 μm, at the edge of the wafer, due to the large Jut-out, a pronounced compression of the pad leads to a distinct load on the wafer, especially at the edge. Meanwhile, as shown in Figure 7a,b, in this contact state, the carrier is not deformed, and only the wafer has a supporting effect on the pad. Therefore, under this contact state, the structure of the carrier, Jut-out, and the size of its gap with the wafer will not have any effect on the wafer stress distribution. Secondly, when 0 μm ≤ Jut-out ≤ 2 μm, as shown in Figure 7(c1,c2,d1,d2), the carrier starts to provide support to the pad on the edge of the wafer, which helps to reduce stress concentration. As the Jut-out decreases, the support effect of the carrier on the upper pad gradually increases. However, due to the existence of the gap between the wafer and the carrier, the pad exhibits a certain amount of deformation within this gap. Although the pad deformation is much reduced compared to the initial stage, its existence still leads to edge stress concentration, resulting in edge roll-off. The illustration is shown in Figure 9iii,iv. Finally, when Jut-out < 0 μm, the edge of the wafer is not stressed as the wafer edge is separated from the upper pad due to the support effect of the carrier on the upper pad, which reduces MRR at the edge and suppresses the wafer edge roll-off, as shown in Figure 7(e1,e2,f1,f2).

Based on the stress and pad deformation analysis above, the DSP process can be divided into six stages, as shown in Figure 9:

(i). Stage one represents the initial process condition where the silicon wafer is in contact with the upper pad but not with the carrier;
(ii). Despite the decrease in the silicon wafer thickness, the compression effect on the upper pad and wafer stress distribution tends to be the same;
(iii). Silicon wafer and carrier begin to contact the pad simultaneously;
(iv). As the silicon wafer thickness continues to decrease, pad deformation and stress gradually decrease;
(v). The silicon wafer edge in contact with the upper pad begins to separate;
(vi). The silicon wafer edge separates from the upper pad.

### 3.2. Comparison with Experiments

**Figure 10:**
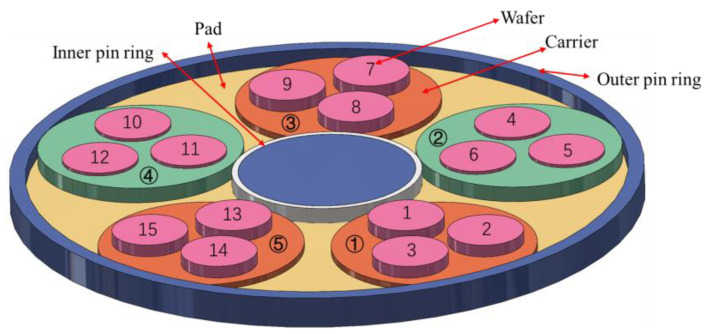
Schematic diagram of the position and thickness of carriers used in the experiment.

**Figure 11:**
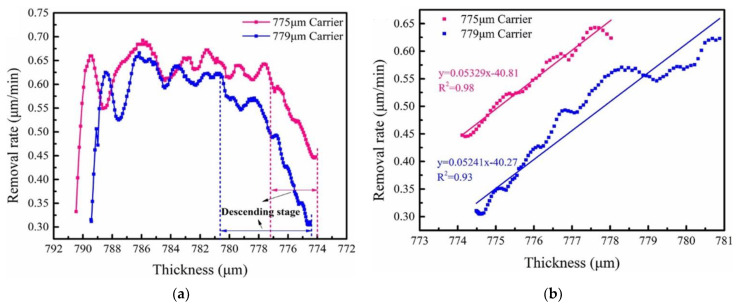
The removal rate of material as a function of wafer thickness with two carrier thicknesses (775 μm and 779 μm). (**a**) The whole process; (**b**) linear fit to the descending stage. (Reprinted from [34], Tao Wei, Yun Liu, Minghao Li, Lei Zhu, Wenjie Yu, Zhongying Xue, Xing Wei, Experimental and numerical analysis of wafer-to-wafer uniformity in double-sided polishing/Influence of the carrier thickness on the wafer-to-wafer thickness variation, Pages No.386, Copyright (2024), with permission from Elsevier [Or Applicable Society Copyright Owner]).

The effect of carrier and silicon wafer thickness on the polishing effect under the same machine and process conditions has been explored by Tao Wei et al. from our team in a previous study [34]. Due to the limitations of the experiment, the effects and impact of each variable cannot be detected separately during the polishing process. Meanwhile, in the experiments, the change in silicon wafer thickness is the only measurable indicator to detect the polishing effectiveness and its causes. We use simulations to provide a detailed mechanistic explanation and validation of the experiments. In this paper, we decompose the dynamic process using FEM to specifically investigate the influence of each variable. The experiment utilizes a commercial DSP machine (Peter Wolters Inc., Rendsburg, Germany, AC2000-P^4^) to produce 300 mm silicon wafers. The AC2000-P^4^ integrates a laser interferometer with an accuracy of ±0.1 μm on the upper pad, which is used to continuously measure the wafer thickness and calculate the real-time MRR. Additionally, a high-resolution wafer geometry measurement tool (KLA Tencor, Milpitas, CA, USA, Wafersight2+™) is employed to measure the wafer thickness after each polishing step. As shown in Figure 10, two different thicknesses of carriers are selected, 775 μm and 779 μm, to investigate the effect of Jut-out on the polishing effect. The polishing process is divided into two stages; the first stage is to polish the initial wafers from 790 μm to 781 μm, and the second stage is to continue polishing to 774 μm. The thickness of each wafer at the end of each stage was measured, and the results are shown in Figure 11.

It can be concluded from the experiments that with the gradual stabilization of the polishing state, the polishing removal rate reaches a stable state. After that, the removal rate of the 775 μm thick carrier starts to decrease around 777 μm, while the removal efficiency of the 779 μm thick carrier starts to decrease around 781 μm. This is consistent with the conclusion drawn from the simulation in this paper that the change in material removal condition appears when the Jut-out is about 2 μm due to the contact between the upper pad and the upper surface of the carriers.

Meanwhile, the support effect of the carriers on the upper pad gradually increases with the decrease in the Jut-out, so that the removal efficiency gradually decreases from about Jut-out = 2 μm. This also verifies the reasonableness of the simulation in this paper.

As can be observed from Figure 11, the removal process can be roughly divided into three stages: initial stage, stable stage, and descending stage. The phenomenon can be further explained by the six contact states derived in Section 3.1. The stable stage in the experiment can be divided into near-contact states and critical contact states to explain the critical value of 2 μm, as mentioned in Section 3.1. Meanwhile, the descending stage is divided into complete contact states, critical separation states, and boundary separation states to further clarify the removal rate decreasing process. Effectively controlling the changes in contact states can efficiently regulate the polishing outcomes.

By appropriately controlling the polishing process, such as increasing the thickness of carriers based on the initial wafer thickness and adjusting the polishing strategy according to the polishing stages, the contact state can be controlled to match stages (iii) to (vi), as shown in Figure 9. This approach helps to reduce stress concentration at the edges of the silicon wafer, thereby inhibiting edge roll-off.

### 3.3. Effect of Pad’s Materials on ERO

**Figure 12:**
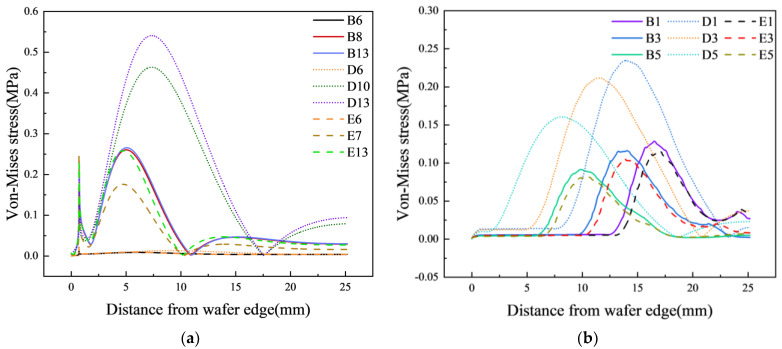
Von Mises stress on the upper surface of the wafer with different Jut-outs. (**a**) Jut-out ≥ 0 μm and (**b**) Jut-out < 0 μm.

To identify methods for improving the contact state, we also conducted simulations using pads made from three different materials to study the impact of pad material properties on stress distribution and to propose possibilities for controlling stress concentration. Figure 12 shows the stress distribution under different pad materials and Jut-out conditions with a 0.5 mm gap.

From Figure 12a, it can be observed that when Jut-out ≥ 0 μm, there are two peaks in the von Mises stress curve on the surface of the silicon wafer. The change in pad material does not affect the position of the first peak. Subsequently, a broader and larger stress peak occurs further from the wafer edge. The change in pad material influences both the magnitude and position, and as the Young’s modulus of the pad increases, both the peak value and width decrease. From Figure 12b, it is also evident that when Jut-out < 0 μm, under the same Jut-out conditions, the pad material continues to affect the stress conditions on the surface of the silicon wafer. The lower the Young’s modulus of the pad, the higher and wider the peak.

**Figure 13:**
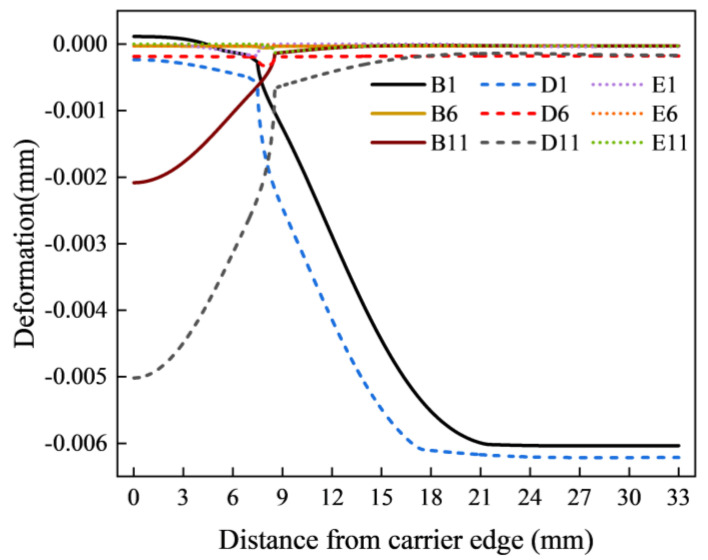
Deformation of the lower surface of the upper pad.

The phenomenon above can be explained by the deformation of the pad depicted in Figure 13. Firstly, when Jut-out > 0 μm, the pad undergoes significant compression. Thus, only a small part of the uniform load transmitted by the pad causes deformation in itself, while a larger portion is transferred to the surface of the silicon wafer. This also exacerbates the degree of pad depression at the wafer edges, where the resultant forces are greater, leading to a greater bending force and a larger peak in equivalent stress on the surface. When Jut-out = 0 μm, the material of the pad does not significantly affect the force on the surface of the silicon wafer. When Jut-out < 0 μm, the lower the Young’s modulus of the pad material, the greater its displacement, resulting in more extensive contact with the silicon wafer surface and, consequently, greater pressure is exerted on the wafer. Therefore, it can be concluded that the lower the Young’s modulus of the pad, the greater the peak.

### 3.4. Effect of Gap Change on Edge Roll-Off

**Figure 14:**
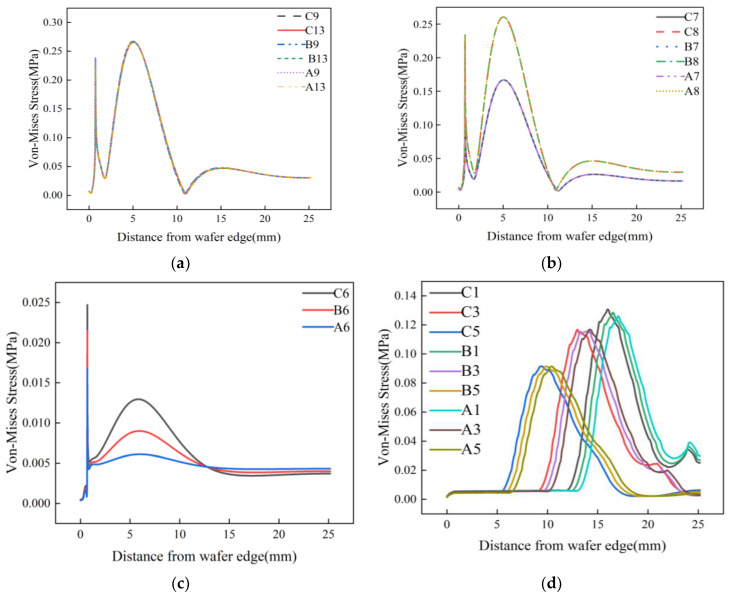
Von Mises stress on the upper surface of the wafer with different Jut-outs and gaps ((**a**) for Jut-out > 2 μm; (**b**) for 0 μm < Jut-out ≤ 2 μm; (**c**) for Jut-out = 0 μm; (**d**) for Jut-out < 0 μm).

In the DSP process, the silicon wafer is not strictly clamped by the carrier, which may cause the gap between them to continuously change, resulting in unstable stress distribution. On the other hand, due to the varying carrier manufacturing tolerances proposed by different manufacturers during the production process, there can be significant differences in the resulting gap [35]. This research conducts simulations considering different gaps and Jut-out values. In this study, three gap sizes are selected: 0 mm, 0.5 mm, and 1 mm. Figure 14 shows the distribution of von Mises stress on the surface of the wafer under different Jut-out conditions for these three gap sizes.

It can be observed from Figure 14a,b that when the Jut-out is greater than 0 μm, the stress curves for different gaps of Jut-out overlap with each other. Although there is a decrease in peak stress values when crossing the critical value, the stress curves for different gaps still overlap under the same Jut-out conditions. As the Jut-out decreases to 0 μm, as shown in Figure 14c, the stress curves for different gaps begin to separate. When gap = 1 mm, both peak values of the von Mises stress curve are the maximum values among the three gap states. When gap = 0 mm, the peak values are the minimum values among the three gap states, and the overall stress curve is smoother. The stress curve for gap = 0.5 mm lies between these two scenarios. When the Jut-out is less than 0 μm, as shown in Figure 14d, increasing the gap causes the peak values of the von Mises stress curve to shift to the wafer center, with no significant differences observed in the stress peak values and curve trends under the same Jut-out conditions, which implies a decrease in the edge roll-off.

**Figure 15:**
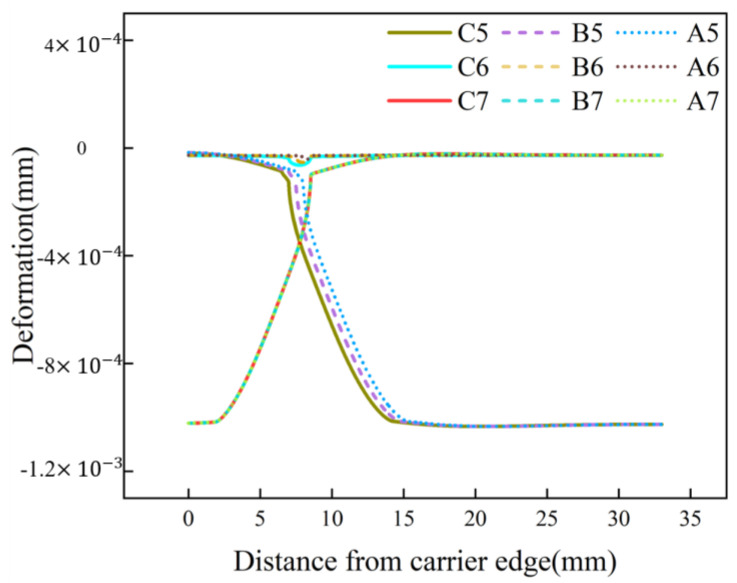
Deformation of the lower surface of the upper pad with different Jut-outs and gaps.

The phenomenon above can be explained by the deformation of the pad and change in contact state, as shown in Figure 15. Firstly, when the Jut-out is greater than 0 μm, the change in gap value does not affect the compression of the pad at the edge of the wafer. Therefore, under this contact state, changes in the gap at the edge of the wafer do not affect the stress distribution on the wafer. Secondly, when the Jut-out is 0 μm, the deformation of the pad at the edge of the wafer escalates with the increasing gap, leading to an increase in the load applied on the edge and a more pronounced stress concentration phenomenon. This change in stress concentration further increases the edge roll-off of the wafer. Lastly, when the Jut-out is less than 0 μm, increasing the gap leads to a larger pad deformation within the carrier. Thus, under the same Jut-out conditions, the contact area between the wafer and pad gradually rises with the increasing gap, providing more sufficient contact with the edge of the wafer and inhibiting the separation of the pad from the wafer edge.

Controlling the range of this gap variation is helpful to improve the wafer quality. Adjusting the fluctuation range of the gap to between 0 and 0.5 mm not only alleviates the stress concentration at the edges of the silicon wafer but also facilitates subsequent operations without impeding the polishing process.

## 4. Conclusions

In this paper, a 2D finite element structural model of the DSP process is established to analyze the entire polishing process, and the result is compared and verified by experimental studies. Firstly, we analyzed the variations in the contact state and stress distribution caused by the changes in thickness of the silicon wafer during the polishing process. Secondly, simulations of different pad materials are conducted to discuss their impact on the stress distribution on the wafer’s upper surface. Additionally, the gap between the silicon wafer and the carrier is investigated to study the impact of the gap on the changing contact state during the polishing process. It can be concluded that the Jut-out directly influences the contact state during the DSP process, thereby defining critical stages. Meanwhile, when the Jut-out is greater than 2 μm, the stress on the wafer edge remains concentrated due to the separated contact state, resulting in no change in the removal rate on the wafer surface as the polishing progresses. A Jut-out between 0 μm and 2 μm reduces the stress concentration at the wafer edge, promoting uniform removal and minimizing edge roll-off. A Jut-out below 0 μm inhibits edge removal, with contact shifting toward the wafer center. It can also be concluded that a harder pad reduces the stress concentration at the wafer edge, thereby further mitigating ERO. In addition, gap variations affect the stress concentration at the wafer edge when Jut-out ≤ 0 μm, impacting edge roll-off. An increased gap exacerbates edge roll-off and prolongs pad separation from the wafer edge. By considering the changes in wafer thickness and the circumferential gap during the polishing process, the forces at the edges of the wafer can be controlled more precisely. Adjustments such as using a harder pad, reducing the inner diameter of the carrier to minimize the gap change at the silicon wafer edge, and using carriers of different thicknesses for stepwise polishing can be adopted to prevent the edges from rolling off. This study provides insights into the mechanism underlying the contact and stress concentration behaviors during DSP and helps to mitigate edge roll-off, enhance stress distribution uniformity, and improve wafer flatness.

## Figures and Tables

**Table 1:** Meaning and magnitude of the parameters in the model.

Parameter	Meaning	Value	Parameter	Meaning	Value
$h_{H}$	Thickness of upper head	1.23 mm	$h_{UP}$	Thickness of upper pad	1.23 mm
$h_{C}$	Thickness of carrier	0.781 mm	$h_{LP}$	Thickness of lower pad	1.23 mm
	Length of carrier	7 mm	$l_{N}$	Length of carrier PVDF	0.5 mm
$r_{P}$	Length of pad	33 mm	$r_{W}$	Length of wafer	25 mm
$r_{D}$	Radius of the chamfering rounded corner	0.27 mm	α	Chamfering angle	21.6°
gap	Distance between PVDF and wafer	0/0.5/1 mm	$h_{w}$	Thickness of wafer	775 μm–795 μm

**Table 2:** Friction coefficient used for frictional contact.

Friction Coefficient Used for Frictional Contact	Friction Coefficient
Upper Pad & Wafer	0.3
Lower Pad & Wafer	0.3
PVDF & Wafer	0.35

**Table 3:** Material properties used in the model.

Part	Young’s Modulus (MPa)	Poisson’s Ratio
Head	5000	0.3
SUBA800M2	205.8	0.2599
SUBA600	34.14	0.2448
SL12	243.25	0.3229
Carrier	193,000	0.31
Wafer	190,000	0.27
PVDF	840	0.3

**Table 4:** Simulation scheme under 1500 daN polishing pressure.

	Gap (mm)	0	0.5	1
Jut-Out (μm)				
−6		A1	B1	C1
−4		A2	B2	C2
−3		A3	B3	C3
−2		A4	B4	C4
−1		A5	B5	C5
0		A6	B6	C6
1		A7	B7	C7
2		A8	B8	C8
3		A9	B9	C9
4		A10	B10	C10
6		A11	B11	C11
9		A12	B12	C12
14		A13	B13	C13

**Table 5:** Simulation scheme under 1500 daN polishing pressure and 0.5 mm gap.

	Pad	SUBA600	SUBA800	SL12
Jut-Out (μm)				
−6		D1	B1	E1
−4		D2	B2	E2
−3		D3	B3	E3
−2		D4	B4	E4
−1		D5	B5	E5
0		D6	B6	E6
1		D7	B7	E7
2		D8	B8	E8
3		D9	B9	E9
4		D10	B10	E10
6		D11	B11	E11
9		D12	B12	E12
14		D13	B13	E13

## Data Availability

The original contributions presented in the study are included in the article, further inquiries can be directed to the corresponding author.
